# Patient Sociodemographic Factors Are Associated with Receiving Point-of-care Ultrasound in the Emergency Department

**DOI:** 10.5811/westjem.21297

**Published:** 2025-05-19

**Authors:** Brandon M. Wubben, Devin Spolsdoff, Karisa K. Harland, Marina Del Rios

**Affiliations:** University of Iowa, Department of Emergency Medicine, Iowa City, Iowa

## Abstract

**Background:**

Point-of-care ultrasound (POCUS) is widely used in emergency medicine (EM) and increasingly throughout healthcare. Prior studies have revealed disparities in the use of imaging in the emergency department (ED) based on sociodemographic factors; however, the association between these factors and POCUS use is unknown. Our aim was to compare the odds of receiving POCUS in the ED based on patient race and ethnicity, language, sex, and type of insurance.

**Methods:**

We reviewed electronic health records (EHR) matched to a departmental POCUS database from November 2021–June 2023 at an academic Level I trauma center. We included ED patients diagnosed with an International Classification of Diseases code mapped to chest or flank pain, who had a cardiac troponin obtained, or had been evaluated as a trauma activation or alert. Our primary outcome was whether a patient received transthoracic echocardiography (cardiac), renal, or focused assessment with sonography in trauma. Predictor variables were race/ethnicity group (non-Hispanic [NH] White, NH Black, Hispanic, other), patient language, sex assigned at birth, and insurance type as recorded in the EHR. We performed descriptive analyses and logistic regression (adjusted odds ratio [aOR], 95% confidence interval [CI]) controlling for body mass index, age, comorbidities, and triage hypotension or tachycardia.

**Results:**

Of the 25,389 ED patients meeting inclusion criteria, 79.5% were NH White, 95.3% listed English as their primary language, 51.5% were female, and 33.4% had private payor insurance. After adjusting for confounding, patients had lower odds of receiving POCUS if they were “other” race/ethnicity as compared to NH White (aOR 0.65, CI 0.42–0.99, *P* = .04), female as compared to male (aOR 0.81, CI 0.69–0.94, *P* = .007), or if they had Medicare (aOR 0.67, CI 0.54–0.84, *P* <.001) or Medicaid (aOR 0.66, CI 0.52–0.83, *P* = .001) as compared to private payors.

**Conclusion:**

Overall, patients of female sex and patients with Medicaid or Medicare had lower odds of receiving point-of-care ultrasound in the ED. Although we did not find a difference in POCUS use among non-Hispanic White, NH Black, and Hispanic patients, patients belonging to other race/ethnicity categories had lower odds of receiving POCUS compared to NH White patients.

## INTRODUCTION

Point-of-care ultrasound (POCUS) is an integral part of evaluation and treatment in emergency medicine (EM) and has been shown to have utility in the diagnosis of cardiac conditions including heart failure and renal disease such as urolithiasis.^1^–^3^ The use of POCUS is also associated with faster treatment for life-threatening conditions including cardiac tamponade, ectopic pregnancy, and hemoperitoneum in blunt abdominal trauma.^4^,^5^,^6^ Little is known about whether POCUS use varies based on a patient’s sociodemographic characteristics. Prior studies of radiograph and computed tomography (CT) use found that White patients and those with private insurance were more likely than patients who were not White or were insured by Medicare or Medicaid to have imaging ordered in the emergency department (ED).^7^,^8^ Because the decision to perform POCUS is largely based on clinical judgment and, therefore, subject to individual biases, we hypothesized that there may be unmeasured inequities in how POCUS is used. In this study, we aimed to compare the odds of ED POCUS use based on patient race and ethnicity, language, sex, and insurance type.

## METHODS

We conducted a single-site, retrospective review of ED encounters from November 2021–June 2023 at an academic Level I trauma center with a three-year EM residency, >50,000 annual patient visits, and over 1,500 annual POCUS examinations and no emergency ultrasound fellowship at the time of the study. The overall ED population served is approximately 75% non-Hispanic White (NHW) and 50% female. Because transthoracic echocardiography (cardiac), focused assessment with sonography in trauma (FAST) including extended FAST, and renal were the most performed POCUS study types, we targeted these studies for inclusion. We queried the electronic health record (EHR) for adult and pediatric ED patients who 1) had a cardiac troponin obtained or 2) were diagnosed with an *International Classification of Diseases*, 10^th^ Modification (ICD-10) code related to chest pain including ST-elevation myocardial infarction (STEMI), non STEMI, stable or unstable angina, other chest pain, chest pain unspecified, syncope, pericardial effusion, cardiac tamponade, blunt cardiac injury, myocarditis, pericarditis, or pulmonary embolism included in the cardiac POCUS subgroup. The ICD-10 codes related to flank pain, including renal colic, nephrolithiasis, renal abscess, pyelonephritis, hydronephrosis, or ureteral stricture, were included in the renal POCUS subgroup, and those who were activated as a trauma surgery alert or activation based on hospital protocol were included in the FAST POCUS subgroup. The ICD-10 codes were mapped to ED diagnoses with the assistance of an ED coding specialist.

We extracted demographic and clinical variables from the EHR using system-level query tools, including age, legal sex (male, female, nonbinary), height, weight, triage vitals (categorized as normal or abnormal for age for hypotension (<90 millimeters of mercury systolic in adults) and tachycardia [>110 beats per minute], ICD-10 diagnoses, comorbidities using the revised Charlson Comorbidity Index (CCI) (analyzed as both comorbidities recorded based on patient problem list ICD-10 codes vs not recorded, and for those with comorbidities recorded, a score of 0 vs >0), and preferred language (English, Spanish, and other). For race and ethnicity, we used a collapsed category methodology similar to Ross et al (NHW, non-Hispanic Black [NHB], Hispanic, and other race).^7^ Insurance type was divided into uninsured, Medicare, Medicaid, and private payors.

**Population Health Research Capsule**

What do we already know about this issue?

*Prior studies raise concern that White, privately insured patients are more likely to receive emergency department (ED) imaging.*

What was the research question?

*We compared the odds of receiving point-of-care ultrasound (POCUS) in the ED based on patient race and ethnicity, language, sex, and insurance type.*

What was the major finding of the study?

*Female (aOR 0.81, CI 0.69–0.94), Medicare (aOR 0.67, CI 0.54–0.84) and Medicaid (aOR 0.66, CI 0.52–0.83) patients had lower odds of POCUS use, compared to non-Hispanic Whites.*

How does this improve population health?

*Disparities in POCUS use illuminate the need for critical review of current practice. Future work might focus on factors contributing to these disparities, and interventions to address them.*

Our emergency ultrasound quality assurance (QA) database was queried for corresponding POCUS exams and matched to EHR records by medical record number and date of ED visit. All POCUS exams are reviewed and scored for quality by emergency ultrasound fellowship-trained physicians. The POCUS images and reports are then stored in an emergency ultrasound QA database. We assessed differences in clinical vs educational POCUS and POCUS quality as documented in the QA database (categorized as quality scores of 1–3 vs 4–5) by patient demographics using chi-squared tests.

We compared the proportion of ED encounters where POCUS was performed, using descriptive statistics. Associations between POCUS and patient demographics were assessed using logistic regression models accounting for age, calculated body mass index (BMI), presence of comorbidities, and abnormal triage blood pressure or heart rate. Secondary analyses included cardiac, FAST, and renal POCUS subgroups. To determine whether our model should include any interaction terms between patient demographics and POCUS while accounting for age, BMI, CCI, and abnormal triage vitals, we employed the BranchGLM package in R for best subset selection using Akaike information criterion (AIC).^9^,^10^,^11^ We used R software (R Foundation for Statistical Computing, Vienna, Austria) for analyses and data management. The study was approved by our institutional review board, and we followed the Strengthening the Reporting of Observational Studies in Epidemiology (STROBE) guidance.^12^

## RESULTS

There were 25,389 patients who met the inclusion criteria; of these, 6,266 were excluded from the analysis due to missing covariates, leaving a full analysis population of 19,123 composed of 15,677 cardiac patients (82%), 3,443 renal patients (18%), and 1,215 trauma patients (6.4%). Total percentages exceeded 100% due to patients meeting multiple inclusion criteria (eg, a trauma alert with a troponin obtained). Among the full-analysis population, most patients spoke English (96.6%), and the mean age and BMI were similar between non-POCUS and POCUS groups (56.9±20.7 vs 57.2±20.8 years) and (30.2±9.5 vs 29.9±8.1 kilograms/m^2^). The number of patients with no comorbidities documented (10,321 [55.9%] vs 382 [57.2%]) and the frequency of CCI scores of zero when comorbidities were documented (6,199 [33.6%] vs 221 [33.1%]) were also similar between non-POCUS and POCUS groups (Supplemental Table 1). Patients who received POCUS were more frequently hypotensive (49 [7.3%] vs 526 [2.9%]) and tachycardic (124 [18.6%] vs 2,774 [15.0%]). Patient sociodemographic characteristics and odds of receiving POCUS are shown in Table 1.

Patients who listed Spanish or other as their language preference had similar odds of receiving POCUS to those listing English (Spanish, odds ratio [OR] 1.16, 95% confidence interval [CI] 0.65–2.08) (other, OR 0.73 [95% CI 0.38–1.42]). Due to small sample sizes for some patient-language subgroups, adjusted ORs and subgroup analyses are not reported. Likewise, POCUS subgroups without statistically significant differences in the setting of smaller sample sizes are shown in Table 2.

Following best subset selection assessing all possible combinations of two-way interactions between patient demographics, we found no meaningful interactions as determined by the Akaike information criterion. There were no significant associations when assessing differences in clinical vs educational POCUS or differences in POCUS quality by patient demographic characteristics.

## DISCUSSION

Overall, we found that for patients presenting to the ED with an indication for echocardiography, renal, or FAST POCUS, being female, or having Medicare or Medicaid insurance was associated with lower odds of receiving POCUS. In addition, patients who were categorized as other race/ethnicity (comprised primarily of patients identifying as Asian, American Indian, Native Hawaiian, and mixed or other race) were less likely to receive POCUS than patients who were NHW. To our knowledge, an evaluation of these inequities in POCUS use has not previously been reported. However, our findings are consistent with disparities that have been previously reported for other ED imaging modalities.^7^,^8^

Prior analysis of a national ED dataset found that patients belonging to any minoritized race/ethnicity group other than White were less likely to get any ED imaging, including radiograph, CT, MRI, or ultrasound (OR 0.84, CI 0.79–0.89) but did not find a difference when isolating the odds of ultrasound ordered from the ED (OR 1.03, .CI 92–1.14).^7^ The same study found that female patients had lower odds of getting any imaging (OR 0.95, CI 0.91–0.99), but did not specifically examine odds of ultrasound.^7^ Likewise, patients with Medicaid and Medicare had lower odds of getting any imaging (OR 0.82, CI 0.76–0.88 and OR 0.87, CI 0.80–0.95, respectively). A national study of pediatric ED patients also noted markedly lower odds of imaging for patients who were NHB compared to NHW (aOR 0.82, CI 0.82–0.83) and fewer ultrasounds (aOR 0.69, CI 0.68–0.70), but it did not specifically look at radiology-performed studies vs POCUS.^13^

Our inclusion criteria were intentionally broad to capture ED patients where the specific types of POCUS examined would likely be indicated based on diagnosis codes. However, this approach could have underestimated disparities in POCUS use if clinicians failed to make the correct diagnosis more frequently in minoritized patient groups; it is possible that POCUS use in patients who did not receive it may have led to a correct diagnosis that would have not otherwise been made.

The downstream consequences of disparate rates of POCUS use are uncertain and are an area for further study, as they may be contributing to larger scale disparities in emergency care. However, we postulate that based on the known benefits of POCUS, such as identification and earlier drainage of pericardial effusion,^1^ lower rates of POCUS use may result in delays in care and more missed diagnoses for certain sociodemographic groups.

## LIMITATIONS

Our findings are subject to the limitations of a retrospective study, including reliance on EHR data for sociodemographic variables. For example, EHR options for self-reported race/ethnicity not identifying as NHW, NHB, or Hispanic were prespecified to be grouped into an “other” category due to small expected sample sizes. While differences in POCUS use for this group were notable, interpreting this finding is more difficult due to this group’s heterogeneity. Second, some POCUS examinations were performed for educational rather than clinical purposes; however, we found no significant change in odds of receiving POCUS when evaluating clinical vs educational ultrasound using a modified sensitivity analysis. Although we adjusted for common reasons that POCUS may be more likely to be performed, there may have been other unmeasured patient-level factors that could have moderated the disparities seen.

As a pilot study, our sample size was not sufficient to conclusively evaluate for disparities in each POCUS subtype. In addition, few FAST examinations were documented despite an institutional expectation of including FAST in the trauma assessment. We believe this was likely due to images not being saved by the performing physician in the context of a variety of factors, including patient acuity and no middleware access for trauma surgery during the study period. Therefore, FAST exams in the current study include only those documented by emergency physicians. Patients who did not receive POCUS could have received alternative imaging; however, there is no direct imaging replacement for echocardiography other than cardiology-performed echocardiology, which is not routinely available in our ED.

## CONCLUSION

Overall, female patients and patients with Medicaid or Medicare had lower odds of receiving point-of-care ultrasound in the ED. Although we did not find a difference in POCUS use among non-Hispanic White, non-Hispanic Black, and Hispanic patients, patients belonging to other race/ethnicity categories had lower odds of receiving POCUS compared to non-Hispanic White patients. Future research might focus on factors contributing to these disparities and developing targeted interventions to address them.

## Supplementary Information





## Figures and Tables

**Table 1:** Frequency of receiving point-of-care ultrasound (POCUS) in the emergency department by patient sociodemographic characteristics, with unadjusted and adjusted odds ratios of receiving POCUS.

Characteristic	No POCUS (N=18,455)	POCUS (%) (N=668)	OR [95% CI]	aOR [95% CI]^a^
Race/Ethnicity				
Non-Hispanic White	15,128	568 (3.62%)	1.00	1.00
Non-Hispanic Black	1,669	50 (2.91%)	0.80 [0.60–1.07]	0.82 [0.61–1.10]
Hispanic	715	27 (3.64%)	1.01 [0.68–1.49]	1.02 [0.69–1.52]
Other	943	23 (2.38%)	0.65 [0.43–0.99]	0.65 [0.42–0.99]
Legal Sex				
Male	9,264	373 (3.87%)	1.00	1.00
Female	9,190	295 (3.11%)	0.80 [0.68–0.93]	0.81 [0.69–0.94]
Insurance Status				
Private payor	5,421	239 (4.22%)	1.00	1.00
Medicare	8,535	286 (3.24%)	0.76 [0.64–0.91]	0.67 [0.54–0.84]
Medicaid	3,509	99 (2.74%)	0.64 [0.50–0.81]	0.66 [0.52–0.83]
Uninsured	328	15 (4.37%)	1.04 [0.61–1.77]	1.04 [0.61–1.77]
Other	662	29 (4.20%)	0.99 [0.67–1.47]	0.90 [0.60–1.36]

a Adjusted for body mass index, age, Charlson Comorbidity Index scores, and hypotension or tachycardia at triage.

*POCUS*, point-of-care ultrasound; *OR*, odds ratio; *CI*, confidence interval.

**Table 2:** Frequency of receiving point-of-care ultrasound in the emergency department by patient sociodemographic characteristics for included subgroups.

	Cardiac POCUS/no POCUS (%)	Renal POCUS/no POCUS (%)	FAST POCUS/no POCUS (%)
Race/Ethnicity			
Non-Hispanic White	404/12,969 (3.12%)	96/2,726 (3.56%)	70/1,001 (6.99%)
Non-Hispanic Black	38/1,440 (2.64%)	7/287 (2.44%)	5/87 (5.75%)
Hispanic	19/572 (3.33%)	8/174 (4.60%)	2/47 (4.26%)
Other	14/696 (2.01%)	5/256 (1.95%)	4/80 (5.00%)
Sex			
Male	264/8,076 (3.27%)	54/1,371 (3.94%)	59/839 (7.03%)
Female	211/7,600 (2.78%)	62/2,072 (2.99%)	22/376 (5.85%)
Insurance Status			
Private payor	156/4095 (3.81%)	50/1400 (3.57%)	34/513 (6.63%)
Medicare	237/8059 (2.94%)	36/1061 (3.39%)	16/243 (6.58%)
Medicaid	62/2702 (2.29%)	24/840 (2.86%)	13/311 (4.18%)
Uninsured	7/253 (2.77%)	2/58 (3.45%)	6/43 (13.95%)
Other	13/568 (2.29%)	4/84 (4.76%)	12/105 (11.43%)

*FAST, focused assessment with sonography in trauma; POCUS*, point-of-care ultrasound.
