# Supplementary material for: Patient Sociodemographic Factors Are Associated with Receiving Point-of-care Ultrasound in the Emergency Department
Source: West J Emerg Med. 2025 May 19;26(3):486–90. doi: 10.5811/westjem.21297 (PMC12208043; doi:10.5811/westjem.21297)
Supplement: Supplementary file 1 [file wjem-26-486-s001.docx]

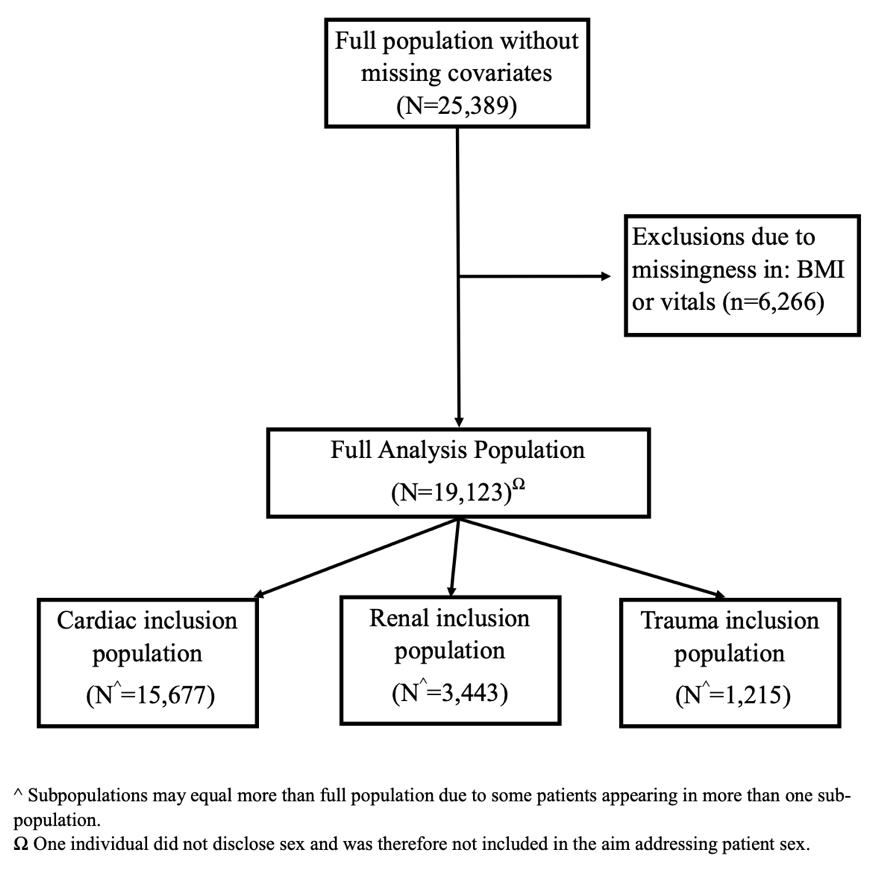


**Figure 1.** Inclusion diagram for emergency department patients meeting inclusion diagnoses criteria. ^ Subpopulations may equal more than full population due to some patients appearing in more than one subpopulation. Ω One individual did not disclose legal sex and was therefore not included in the aim addressing patient sex.
