# Supplementary material for: Patient Sociodemographic Factors Are Associated with Receiving Point-of-care Ultrasound in the Emergency Department
Source: West J Emerg Med. 2025 May 19;26(3):486–90. doi: 10.5811/westjem.21297 (PMC12208043; doi:10.5811/westjem.21297)
Supplement: Supplementary file 2 [file wjem-26-486-s002.docx]

| Characteristic | No POCUS  (N= 18,455) | POCUS  (N=668) |
| --- | --- | --- |
| Language |  |  |
| English | 17,830 (96.6%) | 647 (96.9%) |
| Spanish | 285 (1.5%) | 12 (1.8%) |
| Other | 340 (1.8%) | 9 (1.3%) |
| BMI, mean (SD) | 30.2 (9.5) | 29.9 (8.1) |
| Age, mean (SD) | 56.9 (20.7) | 57.2 (20.8) |
| No Comorbidities Documented | 10,321 (55.9%) | 382 (57.2%) |
| Comorbidity Score >Zero | 1,935 (10.5%) | 65 (9.7%) |
| Comorbidity Score of Zero | 6,199 (33.6%) | 221 (33.1%) |
| Triage Hypotension | 526 (2.9%) | 49 (7.3%) |
| Triage Tachycardia | 2,774 (15.0%) | 124 (18.6%) |

**Supplemental Table 1.** Demographic variables for patients with an indication for cardiac, renal POCUS or FAST based on ED ICD diagnosis or trauma surgery alert or activation.
